# Building capacities in Sub-Saharan African countries for antimicrobial resistance surveillance in the food and agriculture sectors using the FAO ATLASS tool

**DOI:** 10.3389/fvets.2025.1607013

**Published:** 2025-11-03

**Authors:** Labia Irene I. Ouoba, Michael Treilles, Ranyl Nguena Guefack Noumedem, Nicolas Keck, Joshua Kimutai Kiptiness, Alpha A. Diallo, Béatrice Mouillé, Morgane Gourlaouen, Tabitha Kimani, Mark Obonyo, Mamadou Niang, Esther Dsani, Francesca Latronico, Alejandro Dorado Garcia, Emmanuel Kabali, Junxia Song, Mohamed Shamsuddin

**Affiliations:** 1 Regional Office for Africa, Food and Agriculture Organization of the United Nations (FAO), Accra, Ghana; 2 Food and Agriculture Organization of the United Nations (FAO) Headquarters, Rome, Italy; 3 Food and Agriculture Organization of the United Nations (FAO), Nairobi, Kenya; 4 Food and Agriculture Organization of the United Nations (FAO), Dakar, Senegal; 5 Food and Agriculture Organization of the United Nations (FAO), Harare, Zimbabwe

**Keywords:** FAO-ATLASS, antimicrobial resistance, surveillance, laboratory, food and agriculture

## Abstract

FAO has developed the ‘Assessment Tool for Laboratories and AMR Surveillance Systems” (FAO-ATLASS) to support the national food and agriculture sectors in describing and assessing their AMR surveillance system in a standardized manner. Between 2018 and 2024, 221 laboratories and 24 national AMR surveillance systems were assessed in 27 Sub-Saharan African countries. The assessments assigned Progressive Improvement Pathway (PIP) stages from “1-limited” to “5-sustainable,” with stage “3-developed” considered sufficient for reliable AMR data production. The compilation of assessments enabled identification of common gaps that guided FAO interventions to efficiently support capacity building for AMR surveillance in Africa. The impact of the evaluations and follow-up interventions was investigated through a post-ATLASS survey involving 15 of the 27 countries assessed and 112 of the 221 laboratories. The assessments showed that 21 (9.5%) and 6 (2.7%) laboratories were at PIP stages 3 and 4, respectively, while other laboratories (86.5%) are at PIP stage 1 and 2. Two (8.3%) AMR surveillance systems were at PIP stage 3, and others (92.6%) were at PIP stage 1 or 2. Quality assurance was the most critical gap for laboratories; the access to reference strains, reagents, and participation to proficiency testing (PT) scheme were among the major common concerns. For surveillance systems, the data production (laboratory network and data collection/analysis) were the main areas to improve. The post-ATLASS survey carried out in 2024 indicated that over 90% of the countries and 50% of laboratories received support from FAO and partners mainly for training, provision of reagents and equipment (60%), enrolment in PT program (73%), development of AMR surveillance strategy and SOPs on AMR detection (53%). Ten laboratories moved from PIP stage 2 to 3, eight from stage 1 to 2, and two from stage 2 to 4. For about 60% of the laboratories, the respondents reported that the management showed better commitment in supporting AMR activities. Implementation of AMR surveillance is now effective in 80% of the countries surveyed (initial of 33%). The results of this analysis indicate that AMR surveillance systems are progressively improving in African countries; however, sustained efforts are necessary to ensure the production of reliable data in the majority of countries and to inform evidence-based interventions against AMR.

## 1 Introduction

The misuse of antimicrobials can lead to the development of antimicrobial resistance (AMR), a growing threat for the health of humans and animals. The World Health Organization defines AMR as “when bacteria, viruses, fungi, and parasites change over time and no longer respond to medicines, making infections harder to treat, and increasing the risk of disease spread, severe illness, and death” (1). A recent publication on the global burden of AMR estimated that 1.27 million deaths were directly attributed to antibiotic resistance of bacteria only (2).

The difficulty in assessing the trend of infectious diseases in most developing countries is the limited availability of data, a critical area where progress needs to be made to enable effective and sustainable disease diagnostic and monitoring. Surveillance is a crucial process for monitoring trends and patterns, as well as effects of therapeutic and policy interventions. It must be conducted in a systematic manner to provide outcome-specific data needed for planning, implementation, evaluation and overall addressing public health challenges like AMR (3). Countries need evidence-based guidelines to structure the development of effective and efficient surveillance for AMR. Further, harmonized evaluation approaches and tools that are adapted to the complexity of One Health surveillance systems should be developed, integrated into global AMR surveillance, and promoted at the international and national levels (4) as recommended by the One Health joint plan of action 2022–2026 of the quadripartite organizations including the Food and Agriculture Organization of the United Nations (FAO), the World Health Organization of the United Nations (WHO), the World Organization for Animal Health (WOAH) and the United Nations Environment Program (UNEP). Data from various sources are needed to provide a comprehensive and science-based approach for assessing antimicrobial use and resistance in the animal health sector (5) and to complement data in human health and other sectors (6–9). The 2016 FAO Action Plan on AMR identified the necessity for the development of capacity for AMR surveillance and monitoring in the food and agriculture sectors. The new FAO AMR Action plan 2021–2025 was validated with the aim of helping accelerate progress in developing and implementing multi-sectoral national action plans to tackle AMR by calling attention to strategic priorities and areas of expertise for FAO support. As part of this collective effort, the FAO has developed the Assessment Tool for Laboratories and AMR Surveillance Systems (FAO-ATLASS), which is designed to assist countries in conducting a systematic assessment of their AMR surveillance system in food and agriculture (10). The FAO ATLASS tool comprises two distinct modules: one for assessing the capacities of individual laboratories to perform, e.g., microbial identification and AST for surveillance purposes, and another for evaluating the overall capacities of national surveillance systems, taking into consideration the assessments of individual laboratories as well other key components such as governance and sustainability and communication. A detailed description of the FAO-ATLASS tool and its features is provided in the methodology section. The tool can be used to generate baselines, to monitor progress, and to support countries in building their AMR surveillance system in the food and agriculture sectors. Further, it helps and encourages countries in improving AMR surveillance status in a progressive manner, share reliable AMR data at national level and plan for harmonized regional and global AMR surveillance and data compilations for food and agriculture sectors. Additionally, in 2024, FAO launched the International FAO Antimicrobial Resistance Monitoring (InFARM) system to support countries in generating, collecting, analyzing, and utilizing AMR data and evidence for decision-making following international standards. InFARM also provides a mechanism for countries to participate in global AMR integrated surveillance efforts. The information gathered through ATLASS assessments is critical for understanding the reliability of AMR data produced by countries and reported to the InFARM system (11).

Between 2018 and 2024, 221 laboratories from 27 African countries have been assessed using the FAO-ATLASS tool and some interventions based on the assessment recommendations were implemented. The purpose of this study was to collate and synthesize data from the national ATLASS assessments to generate a regional situation analysis in Sub-Saharan Africa for AMR surveillance, specifically in the agri-food sector and assess the efficiency of the FAO-ATLASS tool for capacity building for AMR surveillance. Specifically, the study describes the status of surveillance systems and laboratories assessed with FAO-ATLASS from 2018 to 2024 on their capacity for AMR surveillance to generate reliable data. Further major outcomes of post-ATLASS follow up interventions to support capacity building for AMR surveillance in the countries is depicted. This regional analysis herein reported on different AMR surveillance systems in Africa is anticipated to help better define shared capacity building programs to improve data standardization, such as common AMR indicators and surveillance protocols.

## 2 Materials and methods

### 2.1 Countries and laboratories assessed

**Figure 1:**
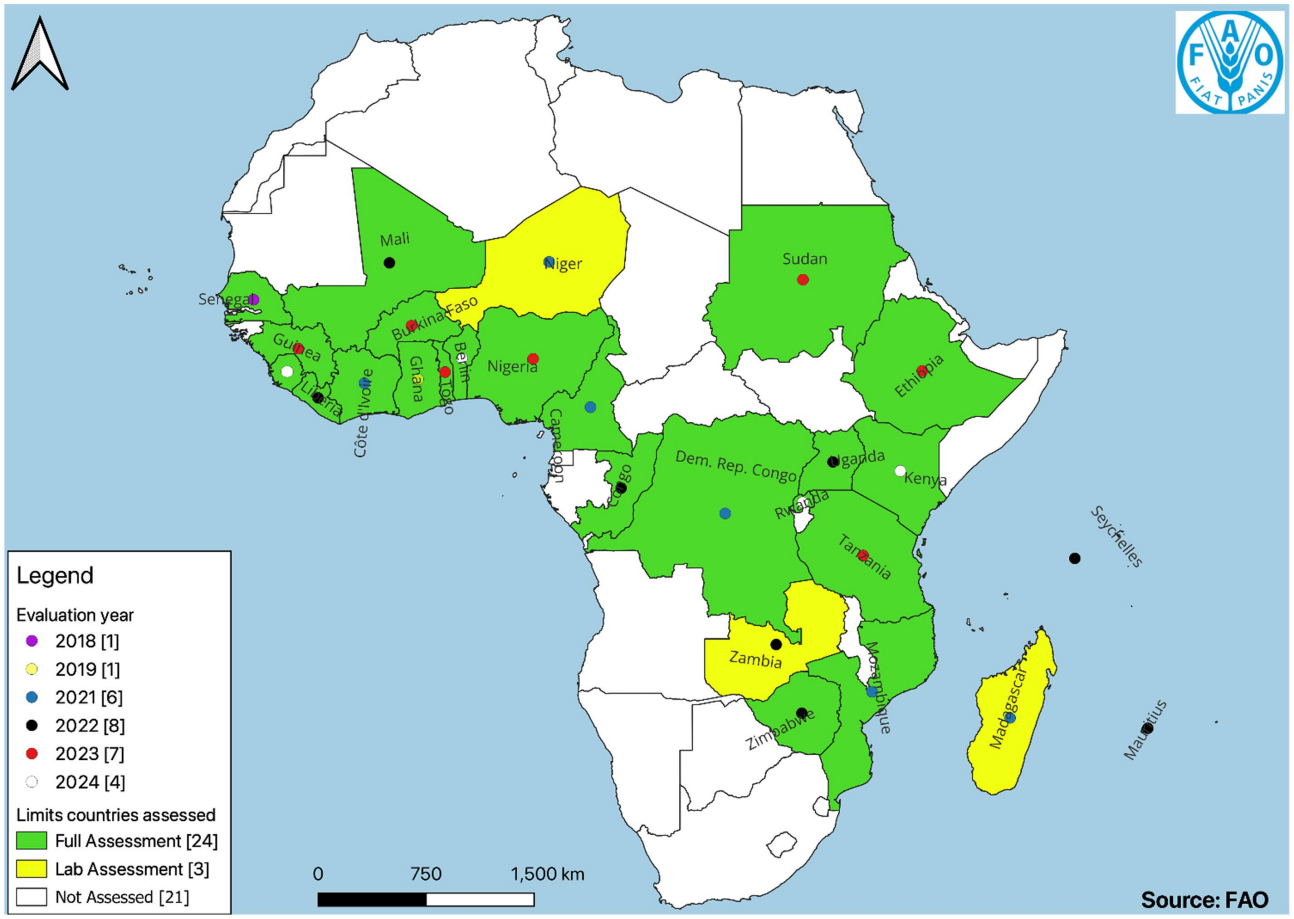
Map of African countries assessed using FAO-ATLASS, 2018–2024. “The boundaries and names shown and the designations used on this map do not imply the expression of any opinion whatsoever on the part of FAO concerning the legal status of any country, territory, city or area or of its authorities, or concerning the delimitation of its frontiers and boundaries” (https://www.fao.org/datalab/dashboard/datalab-maps-disclaimer/).

Assessments of 24 surveillance systems and 221 laboratories from 27 countries (Figure 1) in Sub-Saharan Africa were carried out between 2018 and 2024. The laboratories were from animal health, environment, plant health and food safety including food safety laboratories from the human health sector. For the purpose of this synthesis, 200 laboratories were considered according to the inclusion criteria described in section 2.2.3.

### 2.2 Data collection

#### 2.2.1 The FAO-ATLASS tool

FAO-ATLASS is a tool for assessing and defining targets to improve national AMR surveillance systems in the food and agriculture sectors. It is made of two modules: the surveillance module composed of the Surveillance Evaluation Tool (SET) and the laboratory module composed of the Laboratory Mapping Tool (LMT). Each module includes a descriptive and semi-quantitative questionnaire. The structure of the surveillance module (Table 1) is based on five critical areas of an AMR surveillance system: governance, data production network (laboratories), data collection and analysis, communication, and sustainability, when the structure of the laboratory module (Table 2) is focusing on the following areas: activity, technical practices, management of data and biological material and quality assurance There are two types of FAO-ATLASS assessments, full assessment when the AMR surveillance system and the laboratories are assessed, and Laboratory assessment when only laboratories are assessed.

**Table 1:** Areas and subcategories as defined in the surveillance module of the FAO-ATLASS tool.

Area	Subcategories
Governance	Existence of an operational structure representative of the stakeholders involved in AMR surveillance under One Health approach (multi-sectoral working group(s) or coordination committee on AMR)
	Development of a National Action Plan on AMR involving the food and agriculture sectors
	Relevance of AMR surveillance objectives and AMR indicators in food and agriculture sectors
	Regulations on AMR surveillance organization in the food and agriculture sectors
Data compilation and analysis	Existence of an operational management structure (central epidemiology unit) in food and agriculture sectors
	*Frequency of coordination meetings between central epidemiology unit with local units*
	Representativeness of the surveillance sampling scheme in food and agriculture sectors including environment
	Adequate skill level in AMR epidemiology of members of the central unit
	Adequacy of the data management system for the needs of the AMR surveillance system (database, etc.)
	Data input interval in accordance with the objectives and use of AMR surveillance system results
	AMR data verification and validation procedures formalized and operational
	Analysis of AMR data fits the needs of the system
Data production network	Effective integration of competent laboratories in the AMR surveillance system
	Level of the standardization of work between different laboratories involved in the AMR surveillance system
	Relevance of laboratory diagnostic techniques
	Technical level of AMR data management of the laboratory network
	Frequency of data transmission to the epidemiology unit
	Harmonization of data transmitted to the epidemiology unit
Communication	External policy for communication with decision makers and other stakeholders
	Identification and coverage of key stakeholders’ expectations about the results of the surveillance system
	Existence of awareness building AMR programs for surveillance actors
	Communication of risk assessment outcomes to relevant parties
	Regular release of reports on AMR surveillance results
	Systematic distribution of AMR surveillance results to field actors (outside of a report)
	Presence of a communication system organized between field actors (mail, websites, telephone…)
Sustainability	Adequacy of material and financial resources for the multi-sectoral working group(s) or coordination committee on AMR
	Adequacy of financial resources for the implementation of the National AMR action plan
	Adequacy of human, material, and financial resources for AMR data production (laboratory network) needs
	Adequacy of human, material, and financial resources for AMR data collection and analysis (epidemiology) needs
	Adequacy of human, material, and financial resources for communication needs
	Regular advanced training for actors of the surveillance
	Adequacy of material and financial resources for training
	Development and validation of performance indicators for the AMR surveillance system
	Regular measurement, interpretation, and dissemination of performance indicators
	External assessment carried out
	Implementation of corrective measures

**Table 2:** Areas, categories and subcategories as defined by the Laboratory module of the FAO-ATLASS tool.

Areas	Categories	Subcategories
Activity	Sustainability	Financial capacity (allocation of funds)
		Management
	Workflow organization	Quality of samples submitted
		*Sharing of results with customers*
		Sample acceptation criteria
	Collaborations	Training about antimicrobial resistance
		Scientific publications
		Collaboration with other laboratories in the country
		Collaboration with laboratories outside the country
Technical practices	Resources for bacteriology testing	Biosafety of Bacteriology laboratory
		Equipment for bacteriology and AST
		ANIMAL HEALTH DISEASES - Media and consumable-
		FOOD SAFETY - Media and consumable
		WATER and ENVIRONMENT - Media and consumable
		PLANT HEALTH - Media and consumable
		Reagents availability for AST or identification
	Bacteriology technical practices	Bacteriology methods
		Bacterial identification
	Antimicrobial susceptibility testing (AST) methods	Standard for AST
		Bacterial inoculum calibration for AST
		Panels definition
		Revision of panels of antibiotics
		Method for reading disk diffusion results
		Method for reading MIC results
		Standard for interpretation of disk diffusion results
		Standard for interpretation of MIC results
	Molecular tools	Molecular characterization (resistance gene confirmation or typing)
		Sequencing of resistant strains
Data and strains management	Management of biological material	Sample identification and follow-up
		Proportion of isolates archived in a library
		Method for bacterial preservation
		Inventory of archived isolates
		Duration of bacterial isolates archiving
	Data management	Individual reports on AMR data to the customers
		Data archiving
		AMR data transmission to a dedicated epidemiology unit (if existing) OR STRAINS TRANSMISSION
Quality assurance	Documentation	SOPs on AMR detection implemented OR BACT
		SOPs on AMR detection updating OR BACT
	AMR detection	Reference strains for AST quality control OR BACT
		Proficiency testing for AST OR BACT
	Staff	Initial training in AMR testing OR BACT
		Staff skills validation and continuous proficiency OR BACT

The tool allows, (1) a mapping of national AMR surveillance systems in the food and agriculture sectors, including organization of AMR surveillance and laboratory networks and analytical capacities; (2) a baseline assessment to support the development and implementation of AMR surveillance activities. For that a Progressive Improvement Pathway (PIP) stages are used: 1 “Limited,” 2 “Moderate,” 3 “Developed,” 4 “Demonstrated,” and to “5-Sustainable.” This pathway evaluates laboratories’ capabilities to detect AMR and assesses the ability of surveillance systems to generate and disseminate reliable data on AMR for decision-making processes. PIP stage 3 is considered as the threshold for claiming that AMR data are reliable.

#### 2.2.2 The assessment process

ATLASS assessors followed a training process (initial theoretical training, first assessment under a mentor, second assessment as a lead with report) ensuring standardized assessments from one country to another and from one assessment to another over time. The ATLASS assessment follows a structured process to ensure a comprehensive and standardized evaluation of laboratory and AMR surveillance systems.

Two months before the assessment, the country or relevant authority formally requests FAO to conduct an ATLASS assessment. Upon confirmation, FAO appoints assessors and begins collecting relevant documents for review in preparation for the mission. One week before the assessment, a briefing is held between the assessors and the focal persons from the laboratories to provide an overview of the tool and methodology. On the first day of the assessment, a stakeholder meeting is convened with key stakeholders, including an evaluation of the AMR surveillance system. This is followed by a laboratory assessment, which examines laboratory capacities and operations related to AMR surveillance. On the final day, a restitution stakeholder meeting is conducted to present preliminary findings. Two to four months after the assessment, a detailed report is compiled, reviewed by the FAO team, and shared with stakeholders for input. Following a final FAO review and approval, the report is officially shared with the respective country.

#### 2.2.3 Assessing the status of surveillance systems and laboratories

A MS Excel ® spreadsheet was designed as a data collection tool, to compile and standardize the data from the FAO-ATLASS assessments. The file contains two separate sheets, one for surveillance systems and one for laboratories.

Non-inclusion criteria for laboratories were “laboratories analyzing only human health samples,” “too old versions of ATLASS Excel files (before 2018),” “incomplete file,” “no activity in the laboratory.” Twenty-one (21) laboratories representing a few labs in the 27 countries, were excluded from the analysis for these reasons. For the surveillance systems, data were collected from the countries where both modules (surveillance and laboratory) were used (24 of 27).

All variables of the descriptive questionnaire of the tool were coded as Boolean (Yes/No). For the laboratories or surveillance systems assessed twice, the previous and current results were analyzed to evaluate the Improvement.

#### 2.2.4 Post-FAO-ATLASS survey

To assess the potential improvement and impact of the assessment and follow up interventions to support capacity building for surveillance in the countries, a post-ATLASS survey was conducted. For this purpose, two questionnaires in English and in French were developed (Supplementary materials); one for the laboratories and another one for the surveillance systems. The questionnaires were then digitized using Microsoft Forms ® and the links were sent to the target participants in the different countries.

For ethical considerations, oral informed consent was obtained before participants completed the questionnaire after a thorough explanation of the study objectives. The respondents were informed to have the freedom to reject participation in the study. The participants’ names and all other personal information were handled with confidentiality throughout data collection.

### 2.3 Data analysis

#### 2.3.1 Status of surveillance systems and laboratories

##### 2.3.1.1 Qualitative information

Descriptive analysis was carried out with MS Excel ®. The percentages were automatically calculated as the number of occurrences of a variable value over a total number of occurrences. For laboratories, most variables were converted to Boolean type (Yes/No) to ease the analysis. The map representing the countries assessed was conceived with the QGIS software^1^ version 3.30.0-‘s-Hertogenbosch.

##### 2.3.1.2 PIP stage for laboratories and surveillance systems

The status of the laboratories and surveillance systems were determined by their PIP stages as per the results generated by the LMT and SET questionnaires, respectively. The assignment of the PIP stage is based on the fulfillment of essential components described in each module (Tables 1, 2). Further, the results from the semi-quantitative questionnaires of the modules enabled identification of the gaps and the surveillance components that need to be improved to reach a higher PIP stage. The PIP stages were grouped automatically with a counting formula in MS Excel® and plotted on graphs. The PIP stages were summarized first for the two types of laboratories assessed: “AMR” laboratories which perform both identification and antimicrobial susceptibility testing (AST) and “BACT” laboratories, which perform only bacterial identification. The aspects to improve were highlighted by determining the limiting factor to reach the next PIP stage. The recommendations (subcategories) were weighted “1” or “0.5,” according to whether they are first line priorities (to reach the next PIP stage) or second-line priorities (to reach next+1 PIP stage) respectively, for each laboratory or surveillance system. The principle was that areas with the highest addition of scores should be prioritized for improvement at national level. As the areas of improvement may differ from a laboratory to another and from a PIP stage to another, laboratories were further grouped by PIP stages for the analysis. To avoid an effect related to the number of questions contained in each area, a ratio was calculated by dividing the total score of a subcategory with the number of questions of this subcategory. The Area/Domain with the highest ratio is the priority to consider. A Pareto chart (12) was then constructed for each Area/domain to highlight the most important factors to find the gaps to prioritize to observe the greatest overall improvement for laboratories and for surveillance systems of all countries.

#### 2.3.2 Post-FAO-ATLASS survey

The data collected was analyzed using Microsoft Forms ® online as well as by a descriptive analysis in MS Excel®. French responses were translated into English and the files merged. The next step was to extract and present the general metrics of the responses. The response rate from surveillance systems and laboratories were then analyzed and presented as percentages. Only questions with a response rate above 60% were further considered in the analysis. For each item, percentages were calculated by dividing the number of occurrences by the total number of responses related to the category. Unique choice questions percentages were calculated as the number of respondents who had chosen a given response divided by the total number of respondents to the question; multiple choices questions where recoded into Boolean (Yes/No) type questions, and the percentages for a choice were calculated as the number of “Yes” to this choice on the total number of responses for the question. Open-ended questions (and comments) were analyzed using the integrated text analysis tools of Microsoft Forms®. This has permitted to generate weights for specific words and expressions, based on their occurrence rate.

## 3 Results

### 3.1 Situation of AMR detection and surveillance in Sub-Saharan Africa between 2018 and 2024

#### 3.1.1 AMR surveillance systems

The 27 countries included this analysis are depicted in Figure 1. These represent 56% of the 47 countries from 4 subregions of Sub-Saharan Africa. Out of the 27 countries assessed, 11% were from Central Africa, 33% from East Africa, 41% from West Africa and 15% from Southern Africa.

Taking the surveillance criteria assessed individually, the following strong points can be highlighted: a national action plan was developed in 69% of the countries or under development (18%), an AMR multi-sectoral working group or coordination committee is established in almost 95% of the countries and AMR is generally a concern (awareness) for most of the governments (66%). There are linkages with human health (Policy-legislation, surveillance design, laboratory testing or data analysis) in 58% of the countries. Also, about 58% of the countries AMR data are treated in a central data coordination structure which is most of the time part of an existing “operational management structure (central epidemiology unit)” or contains trained people dedicated to AMR data collection and analysis.

However, key gaps were identified. Less than 30% of the surveillance systems assessed have good data collection/analysis, data production/laboratories, communication, and sustainability scores. Laboratory networks for AMR detection and surveillance exists in 42% of the countries and cover the animal health, human health (food safety component), and environment sectors. However, most of the surveillance systems assessed (66%) were not formally set up and there was no effective integration of competent laboratories in the AMR surveillance system.

Of the 24 countries assessed with the surveillance module of the FAO-ATLASS tool; only two had their AMR surveillance system at PIP stage 3 from the initial assessment. The majority (81%) were at PIP stage 1. The pareto charts prioritize the factors to improve in different domains of the AMR surveillance systems. The three (3) main areas that required improvement are data production network, data collection/analysis and Governance (Table 3).

**Table 3:** Top three limiting factors within relevant area for all AMR surveillance systems assessed in African countries, 2018–2024.

Areas	Main limiting factors
Data compilation and analysis	Data input interval in accordance with the objectives and use of AMR surveillance system results
	Existence of an operational management structure (central epidemiology unit) in food and agriculture sectors
	AMR data verification and validation procedures formalized and operational
Data production network	Effective integration of competent laboratories in the AMR surveillance system
	Frequency of data transmission to the epidemiology unit
	Harmonization of data transmitted to the epidemiology unit
Governance	Relevance of AMR surveillance objectives and AMR indicators in food and agriculture sectors
	Existence of an operational structure representative of the stakeholders involved in AMR surveillance under One Health approach (multi-sectoral working group(s) or coordination committee on AMR)
	Regulations on AMR surveillance organization in the food and agriculture sectors

#### 3.1.2 Laboratory capacities for AMR detection

The following synthesis considers the 200 laboratories which fitted the inclusion criteria. Most of the laboratories are multipurpose institutions. Activities cover animal health, human health (food safety component), environment, and plant health. Food safety is the most covered sector (40%), followed by animal health (31%) and environment (26%). Plant health activities are carried out by 3% of the laboratories. Laboratories performing both microbial identification and antimicrobial sensitivity testing (AST) represent 90.5% of the laboratories assessed and those involved only in microbial identification represent 9.5%.

**Figure 2:**
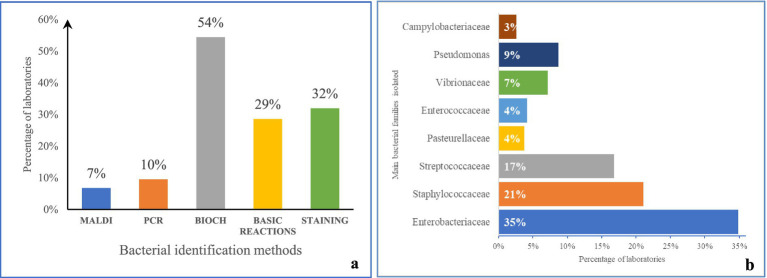
Bacterial identification methods used **(a)**, and main bacterial families isolated **(b)** in the laboratories assessed, 2018–2024. MALDI, Proteomic profiling; PCR, Polymerase Chain Reaction; BIOCH: biochemistry tests (e.g., oxydase, catalase); Basic reactions, Basic color reactions.

Technical practices were assessed and concern mainly methods for bacteria identification (Figure 2), AST and standards. Biochemical reaction including Analytical Profile Index (API) identification is used by most laboratories (54%). Molecular and advanced techniques such as polymerase chain reaction (PCR) and matrix-assisted laser desorption/ionization (MALDI) are used in less than 20% of the laboratories, which most of the time are reference/research laboratories. Basic reactions and staining (Gram mostly) are used but as a step for the early discrimination of bacteria in 32% of the laboratories.

Enterobacteriaceae (*Escherichia coli* and *Salmonella spp*) are isolated in almost all the laboratories evaluated. Two other major families of interest were *Staphylococcaceae* and *Streptococcaceae* generally associated with clinical samples. More than 50% of the laboratories use Disk Diffusion for AST with 35% using an up-to-date international standard for AST: CLSI (19%), EUCAST (16%). Where performed, in general, animal health laboratories use mostly CLSI (57%) and EUCAST (53%) is used for human health pathogens. Most (65%) of the laboratories do not use these two international standards for AST. In these cases, activities are based on old medical bacteriology activities guidelines or protocols and books. Less than 1% of the laboratories use sample inoculation on selective media for specific AMR phenotypes. Automated systems for identification or AST are rare and even when available, they are not used routinely.

With regards to quality control, 29% of the laboratories assessed use reference strains for quality control of AST, mostly *Escherichia coli ATCC 25922* strains. Also, 50% of the laboratories carry out culture media quality controls (sterility and fertility mostly) and 35% of them participate to proficiency testing on AST which is a critical requirement to reach the PIP stage 3.

**Figure 3:**
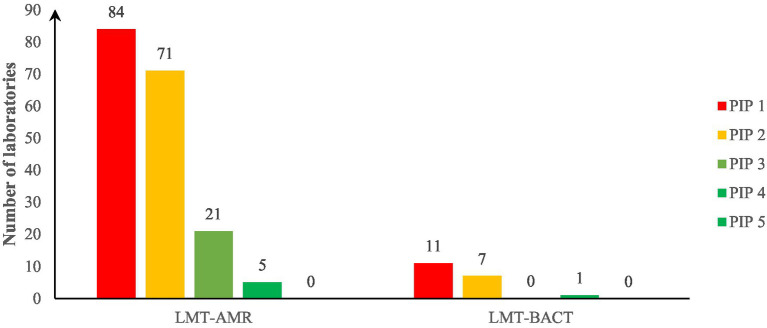
Progressive Improvement Pathway (PIP) stages of laboratories assessed in Sub-Saharan Africa, 2018–2024. LMT, Laboratory mapping tool; AMR, for laboratories performing antimicrobial susceptibility testing; BACT, for laboratories performing only bacterial identification.

Figure 3 shows that 21 (11%) and 5 (2.5%) laboratories were at PIP stage 3 and 4, respectively, in “AMR” laboratories. One (1) “BACT” laboratory has reached the PIP stage 4. The rest of the laboratories assessed were at PIP 1 or 2, both for “AMR” and “BACT” laboratories (see paragraph 2.3.1.2).

For PIP stage 1 and PIP 2 laboratories, areas to improve were related to technical practices such as antibiotics panel definition and revision, use of standards for AST results interpretation and quality assurance component such as proficiency testing for AST or bacteriology, the use of reference strains for AST quality control or bacteriology and AMR detection Standard Operating Procedures (SOP) updating.

The improvements required for PIP stage 3 laboratories to move to the next level were mainly related to some quality assurance components, which was a recurrent limiting area for all the laboratories, independently from the PIP stage. Data and isolates management were also an important issue specifically with AMR data or isolates transmission to a dedicated epidemiology unit. The situation is quite the same for PIP 4 laboratories.

The main three (3) limiting factors by area are summarized in the Table 4. “BACT” laboratories exhibit more management issues than “AMR” laboratories whose issues are mostly related to technical practices.

**Table 4:** Top three limiting factors within each area for all laboratories assessed African countries, 2018–2024.

Areas	Main limiting factors
Activity	Sample acceptation criteria
	Quality of samples submitted
	Financial capacity (allocation of funds)
Technical practices	Revision of panels of antibiotics
	Reagents availability for AST or identification
	Panels definition
Data and biological material management	Data archiving
	Individual reports on AMR data to the customer
	AMR data transmission to a dedicated epidemiology unit (if existing) or strains transmission
Quality assurance	Proficiency testing for AST or bacterial identification
	Reference strains for AST quality control or bacterial identification
	Staff skills validation and continuous proficiency or bacterial identification

### 3.2 Impact of FAO-ATLASS assessments and follow-up actions

The results presented in this section are from the AMR surveillance systems and laboratories surveyed after their last FAO-ATLASS assessment. With regards to the surveillance systems, a total of 15 countries representing 63% of the countries assessed responded to the survey. These included Burkina Faso, Cameroon, Côte d’Ivoire, Democratic Republic of Congo, Ethiopia, Ghana, Guinea, Liberia, Madagascar, Mauritius, Mozambique, Senegal, Sudan, Togo, and Uganda. A total of 112 laboratories from 24 countries responded to the survey, corresponding to a response rate of 64%.

With regards to the post-ATLASS status of the surveillance systems, most of the countries surveyed (87%) have an AMR surveillance system. Activities of these surveillance systems cover animal health (87%), human health (80%), food safety (60%), environment (47%) and plant health (33%). For the remaining countries, there is an intention to set up AMR surveillance in the future when funds become available. A national AMR surveillance strategy/program/plan integrating components and implementation of activities across different AMR surveillance programs in food and agriculture exists in 80% of the 15 countries with an AMR surveillance strategy approved by the government for 67% of them although availability of funds to sustain the activities need to be addressed. Most countries apply passive surveillance (from clinical samples submissions) for sample collection in the framework of integrated AMR surveillance system. Active surveillance with an epidemiologically defined sampling framework is applied by almost 50% of the countries. Sentinel or targeted methods with specifically chosen sites is applied in 27% of the countries. However, only 20% of the countries have a formal epidemiology unit dedicated to AMR, or dedicated people for AMR data treatment in existing epidemiology unit.

**Figure 4:**
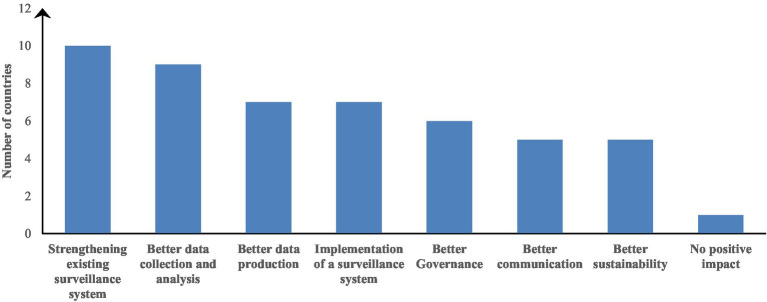
Areas where a positive impact was observed from the FAO ATLASS assessment of the national surveillance systems.

About 93% of the countries assessed received a FAO-specific support after FAO-ATLASS assessment: 12/15 countries received training on bacterial culture and AST as well as AMR data management; 11/15 received reagents and were enrolled in External Quality Assessment (EQA) program; 9/15 received laboratory equipment; 8/15 received support for the development of an AMR national surveillance strategy and the development of SOPs. The support received allowed improvement of the surveillance systems in some areas as described in Figure 4. The area where a positive impact was observed (perceived impact by the respondent to the questionnaire) for most of the surveillance systems is the strengthening of the existing surveillance system (10/15 countries) and the improvement of data collection and analysis (9/15 countries).

From the initial FAO ATLASS assessments, there were only five (5) countries implementing AMR surveillance. The resources provided allowed the implementation of AMR surveillance in an additional seven (7) countries. The implementation of AMR surveillance activities is now effective in 12 of the 15 countries that responded to the survey.

Almost all the respondents (87%) agreed with the PIP stages that was determined by the FAO-ATLASS evaluation. They mentioned that the tool is efficient in capturing the weaknesses and show the gaps, allowing to prioritize actions for improvement. They agreed that the scores obtained reflected their national system conditions. For the systems assessed twice (20%), although there was improvement in governance, recommendations were still made to improve components such as epidemiology, laboratory, communication, and sustainability. The assessment has facilitated the AMR surveillance integration with the One Health approach.

For the laboratories, more than half of the laboratories (69%) which responded to the survey are central laboratories and 73% are involved in both microbiological Isolation/identification and AMR detection. The main purpose (or intended purpose) of AMR detection in 75% of the laboratories is for surveillance. About 70% of them do it as part of diagnostic activities and 63% carry out AMR detection for research purposes. A slight number of laboratories (7%) are training centers and reference laboratories for quality control and epidemiological investigations. More than 85% of the laboratories surveyed support AMR surveillance in their countries, and most of them (62%) are AMR surveillance sentinel/field laboratories.

**Figure 5:**
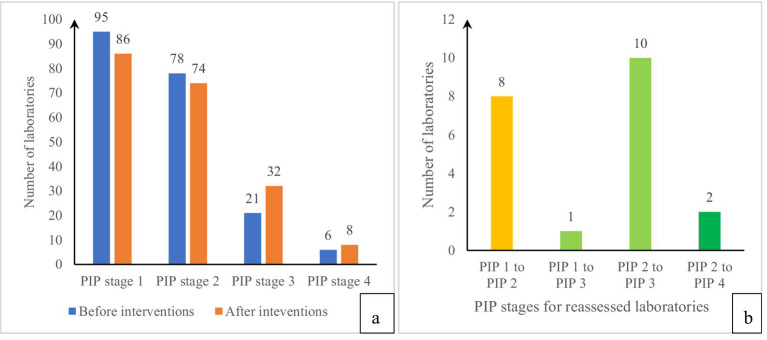
PIP stage evolution **(a)** before and after interventions, and **(b)** for the reassessed laboratories, 2018–2024.

Thirty-six (18%) of the laboratories included in the present study were reassessed (2 to 3 assessments) between 2018 and 2024. The PIP stages for laboratories after and before interventions as well those for laboratories reassessed are shown in Figure 5.

The PIP stages of 19% of the laboratories have increased after FAO-ATLASS evaluation and follow up interventions: 10 laboratories have moved from PIP 2 to 3 as a result of enrolment in proficiency testing scheme; eight (8) have moved from PIP 1 to PIP 2 after training support and reagents provided by FAO and other partners with some of those laboratories being able to increase their number of activities and improve the skills of their technicians; two laboratories have moved from stage 2 to stage 4.

**Figure 6:**
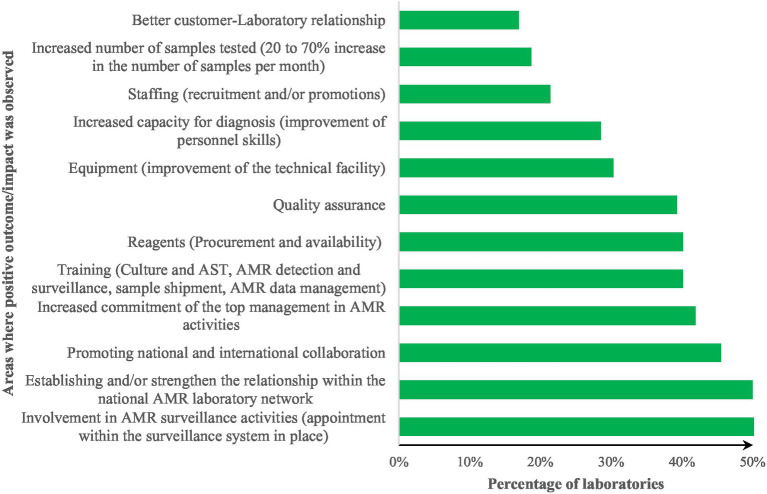
Areas where a positive outcome/impact was reported in the laboratories from FAO-ATLASS assessments and follow up interventions.

A total of 55 of 112 laboratories that responded to the survey received support from FAO or other partners after the FAO-ATLASS assessment to address the gaps identified. The kind of support received includes training, reagents and equipment mostly (42, 34 and 27% respectively). Some laboratories were supported for field missions to increase their level of activities. The areas where a positive outcome/impact was observed in the laboratories from FAO-ATLASS assessments and follow up interventions are represented in Figure 6.

The training supports were specifically on culture and AST (100% of the laboratories that received support), AMR detection and surveillance (80% of the laboratories), infectious disease samples shipment (20% of the laboratories), AMR data management and analysis (40% of the laboratories). Equipment (in 30% of the laboratories surveyed) includes VITEK machines and laboratory renovation (about 10% of the laboratories), small equipment for bacteriology and AST (such as densimeter, balance, pH meter) for 60% of the laboratories. Quality assurance improvement was observed in 39% of the laboratories, particularly with improving SOPs, writing quality manual, enrolment in a PT scheme. The PT includes 50% of the laboratories (for an initial percentage of 35% before support), use of international standards and reference strains for AMR detection. One (1) laboratory has obtained the ISO/IEC 17025:2017 accreditation and another laboratory is working toward ISO 9001:2015 certification and the ISO 17043:2010 accreditation.

For 56% of the laboratories, there is enhancement of top management confidence and commitment on AMR surveillance activities including great support with funds for 25% of those laboratories. More than 80% of the laboratories would like to be reassessed using FAO-ATLASS tool.

Most of the laboratories surveyed agreed with the PIP stages determined by the FAO-ATLASS and found the recommendations very useful. These allow the laboratories to target specific gaps, design a road map, maintain continuous improvement. Further, this was used for advocacy for funds or other support raising. The laboratories are confident that if FAO-ATLASS assessment recommendations is fully implemented capacity of the laboratories will improve, not only in AMR detection and surveillance, but also in improvement of the whole workflow.

## 4 Discussion

The FAO action Plan for AMR 2021–2025 (13) include five main objectives aiming at supporting efficiently AMR mitigation in the food and agriculture sectors. One of the key objectives addresses surveillance which is crucial to generate data. These data will guide decision and policies makers as well as key stakeholders to put in place the best strategies to slow the emergence and spread of AMR strengthening thereby food security and global health. AMR surveillance helps to collect risk-based epidemiological data for specific agri-food value chains and enable a timely assessment and identification of risks before they become large-scale emergencies. To help generate reliable AMR data in the food and agriculture sectors, FAO has developed different tools, initiatives and protocols, one of the key being the FAO ATLASS^2^ (10). The latter provide progressive improvement pathway (PIP) stages designed to assist policymakers in prioritizing actions for building reliable national AMR surveillance systems (9, 10, 14). Conducting an FAO-ATLASS assessment complement results from the human health sector by providing a more global picture of the performance of a country in terms of AMR surveillance including the animal and environmental sectors (9).

With regards to the national AMR surveillance systems assessed, it was noted that most were still in the process of putting in place a functional system although some AMR surveillance activities exist in some countries. It is worth highlighting the importance of the existence in most countries of an AMR national action plan (NAP) and a multisectoral national AMR working group/committee, some of which include a technical working group on surveillance. The WHO global action plan developed with the support FAO and WOAH (15) highlight the importance of developing an AMR NAP as an initial step for establishing an efficient AMR surveillance. AMR NAP indicates the recognition of AMR as a matter to address in the countries and guide context-specific AMR response (16, 17). Further, AMR NAP and multisectoral AMR committees support better coordination and resource mobilization for the efficient implementation of AMR mitigation activities including the surveillance component. The use of a One Health approach is critical in ensuring sustainability of the collaborative, multi-sectoral, and transdisciplinary efforts at the local, regional and national level (4, 18). However, some AMR NAPs do not include specific sectors such as plant and aquaculture and this needs to be addressed in revised AMR NAPs to support better surveillance activities across sectors. Also, some AMR committees do not include key stakeholders such are farmers and the meeting frequency or the lead for the coordination need some improvement (16). This is most of time related to lack of funds dedicated to the AMR working group secretariat and advocacy needs to be made at government level in most cases to support the AMR committees. In more than half of the sub-Saharan countries assessed in the current study, AMR surveillance data are most of the time handled mainly in existing epidemiological units in ministries where one or two people are trained on AMR data management to assure the collection of AMR data from the laboratory network. The absence of a dedicated epidemiology for AMR data is not an issue as such if there is an efficient system in the existing units that ensure a proper management of the AMR data. The problem lies more in the lack of harmonized procedures and specific skills in many cases which render difficult the standardization of the data produced, thus making global data analysis fastidious and sometimes impossible.

An additional good point is that the assessments showed clear links between food and agriculture systems and human health, particularly in terms of policy, legislation, surveillance design, laboratory testing, and data analysis. These links could potentially allow for a more integrated approach to AMR surveillance data in those countries, which might facilitate a multisectoral handling of AMR.

One key gap relates to the fact that more than half of the surveillance systems were not formally set up with clear terms of reference. It is most of the time groups of laboratories which decide on a commitment to work together and share AMR data. Among other factors, this situation contributes to the lack of efficient and harmonized data production, collection, analysis and communication observed through the assessments. Most of the laboratories in the surveillance system are dominated by governmental human health/public health laboratories followed by those of animal health. The private sector and research labs are most of the time not considered sufficiently whereas they may have better capacity to support the national surveillance activities. Formal network of laboratories in food and agriculture sector is limited although effort have been seen in a few countries to put in place and/or strengthen a network in a view of integrating or collaborating with those of the human health sector. Yet, such network when it exists, is dominated by veterinary laboratories, with plant and environment left out most of the time. The limited availability of AMR surveillance networks in the food and agriculture sector especially in low- and middle-income countries has been reported before (19). The report of the second-round results of the quadripartite AMR country self-assessment survey showed a significant difference between the food and agriculture and environment sectors with the human health sector where most countries have established a functional AMR surveillance system for common bacterial pathogens. With regards to the 2024 data of the Tracking Antimicrobial Resistance Country Self- Assessment Survey (TrACSS)^3^ 88% of the African countries reported at least a “limited” surveillance in the human health sector. On the non-human side, 76% of the countries collect some data from animal (Terrestrial and aquatic), 69% from food and 47% from plants whereas only 12% of countries have ongoing surveillance activities in the environment sector.

To help support better capacity building for AMR surveillance in the food and agriculture sector, FAO has developed, the International FAO Antimicrobial Resistance Monitoring (InFARM) system (11). InFARM assist countries in collecting, collating, analyzing, visualizing, and effectively utilizing their AMR monitoring and surveillance in livestock, fisheries, aquaculture, and the plant sectors. The first open call for data was launched in 2024 inviting countries to participle in regardless of their status for AMR surveillance implementation status (Participate | Antimicrobial Resistance | Food and Agriculture Organization of the United Nations). After closing the call for data in 2024, a total of 50 countries participated globally with 20 countries from the Sub-Saharan Africa subregion. This can be attributed to a regional training of focal people from 24 countries in sub-Saharan Africa done in July 2024 in Zambia and strong advocacy of FAO regional, subregional and countries offices to support the initiative (FAO Empowers National Focal Points with Training on Antimicrobial Resistance Surveillance |Antimicrobial Resistance|FAO). The participation of countries in the InFARM system, will help build efficient national AMR surveillance systems in the food and agriculture sectors and help generate high-quality AMR surveillance data, supporting thereby evidence-based actions to address AMR in the countries and the continent. The FAO ATLASS tool is a corner stone for InFARM because it is the mechanism to assess the level of reliability of AMR data reported into the system. InFARM builds on prior collective experience and knowledge gained by FAO and Quadripartite organizations through the implementation of activities on surveillance capacity building with the provision of guidelines and materials. InFARM acts as the bridge for integrating AMR data in animals, food and plant with information from the WHO Global Antimicrobial Resistance and Use Surveillance System (GLASS) and the WOAH ANImal antiMicrobial USE Global Database (ANIMUSE) into the Quadripartite Global Integrated System for Surveillance of Antimicrobial Resistance and Antimicrobial Usage (GISSA). The latter platform is currently under development and will offers better prospects for global and integrated AMR surveillance as the One health surveillance added value to increase the performance of AMR surveillance and, ultimately, improve health outcomes.

With regards to the assessment of the laboratory capacity for AMR detection it was noted that in general, laboratories were capable of isolating and identifying usual surveillance microorganisms such as enterobacteria and perform AST using biochemical methods and applying the minimum required quality controls. This is supported by the TrACSS 2024 data^4^, which showed 49% of the laboratories included in the AMR surveillance system in Sub-Saharan Africa countries use relevant diagnostic techniques for AMR. The use of molecular biology and advanced techniques (PCR and MALDI) for AMR detection and characterization in a few references and/or research laboratories support better microbial identification and susceptibility profiling (20) although the availability of the latter was limited. Some laboratories were assessed toward their capacity in detecting AMR, but a few were evaluated for bacterial identification. In fact, a laboratory may not have a good AST capacity or AST may not be a target for them but be excellent in bacterial identification. Those laboratories can play a key role in ensuring a sound identification of the bacteria in the surveillance systems and then transmit the samples to other laboratories (e.g., the national/sectoral AMR reference center) for determining the AST patterns and analyzing the data.

It was noted in the assessments that *Enterobacteriaceae* (*Escherichia coli* and *Salmonella spp*) were isolated in almost all the laboratories evaluated, due to their relative ubiquitousness and their importance as AMR surveillance indicators. Bacterial families isolated by the laboratories assessed correspond to the general tendency observed in many studies across the African continent. In most cases, surveillance of antibiotic-resistant bacteria in the food and agriculture sector target pathogenic bacteria, such as *Salmonella* spp. and *Campylobacter* spp. as well as indicator bacteria, such as *E. coli* and *Enterococcus* spp. (16, 21–23).

Some laboratories challenges observed in many cases relate to weak laboratory infrastructure, sustainable availability of diagnostic reagents and consumables and insufficient fund to sustain surveillance activities. It has been reported earlier that studies on AMR on bacteria from food animals in several developing countries especially are limited mostly because of under-resourced laboratories (23). It is important to have quality laboratory settings for efficient surveillance. However, there is an issue for sustainable surveillance as most labs rely on external/ project funds with limited time for surveillance activities and little regular internal funds as also reported earlier (24).

The areas of improvement in laboratories differ from one PIP stage to another. PIP 1 and PIP 2 would be weak mostly in activities, technical practices, data and strains management and Quality Assurance (Proficiency Tests), whereas PIP 3 to PIP 5 laboratories would have issues mostly in data and strains management and quality assurance. In fact, the number of factors to improve from PIP 3 to PIP 4 and from PIP 4 to PIP 5 are 13 and 9, respectively, and most of them are related to quality assurance and data and strains management (10). For many laboratories, the EQA/Proficiency testing program was a crucial limiting factor to reach PIP 3. This has been reported before as a general trend in Africa by Okolie et al. (16). The lack of funding and accessibility to an EQA provider were reported as the mains causes. It is important that when assisting a country for AMR surveillance, financial and technical partners make provision for EQA. This allows individual laboratories to identify issues in their practices and formulate appropriate corrective actions to help generate valuable and reliable data.

The post-ATLASS survey showed that surveillance of AMR is now established in almost all the countries assessed and that responded to the survey, as a result of the FAO-ATLASS recommendations and follow-up interventions by FAO and different partners. Further, for countries with existing surveillance system the capacity was strengthened. A better data collection and analysis was observed as a result of training provided to specific people in the existing epidemiology units on AMR surveillance. Laboratories were able to improve their infrastructure, technical capacities and level of activity. Some of the laboratories are working toward accreditation for specific protocols and also meet the requirement of and AMR reference laboratory. In East Africa FAO has supported the development and publication of AMR monitoring and surveillance guidelines for food-producing animals and their products to support harmonization of surveillance implementation in the countries (reference). The document is used in East African countries to help harmonize AMR surveillance in the animal health sector in the subregion. Further, it will serve as the basis for the development of similar guidelines for the other subregions in Sub-Saharan Africa. These positive outcomes observed from the post-ATLASS interventions clearly shows that FAO ATLASS is a valuable tool that efficiently support identification of AMR surveillance gaps and associated corrective actions to help generate science-based evidence. It further serves as a resource mobilization tool for surveillance as the recommendations are sound justification to obtain funding to support AMR surveillance.

Aenishaenslin et al. (25) and Sandberg et al. (26) reported that among many evaluation tools for AMR surveillance, FAO ATLASS is the most user-friendly tool, valuable for risk managers and useful for assessment and improvements in a progressive manner. Other evaluation tools such as NEOH (Network for Evaluation of One Health) and ISSEP (Integrated Surveillance System Evaluation Project) were perceived as the best tools for evaluation of One Health aspects, and ECoSur (Evaluation of Collaboration for Surveillance) as best for evaluation of the quality of collaboration (26). FAO is currently working on a digitalized version of ATLASS to be more user friendly and this will also allow easier compilation of assessment data.

The results of the FAO-ATLASS assessments and post-ATLASS survey herein reported clearly show that surveillance activities for AMR are improving in Africa. However, there is still considerable variation in the implementation of various core components as reported earlier (14, 22, 25, 26, 27, 28) and harmonization at national and regional level is required. Regular funding including countries internal funds are crucial for sustainable implementation of AMR surveillance activities and advocacy needs to be made by national AMR committees for integration of AMR in ministries budgets.

## 5 Conclusion

FAO-ATLASS is an accurate tool for AMR surveillance gaps detection, that could help governments and technical and financial partners to direct their resources straight to the most relevant aspects. The results showed that the interest for the implementation of AMR surveillance activities has increased and most of the surveillance systems have been strengthened after follow-up interventions. AMR surveillance strategies are available in the countries, but sufficient funds are not allocated for activities. Laboratories in general found the recommendations of FAO-ATLASS evaluations to be very relevant and useful. These have permitted them to address specific gaps in AMR or bacteriology activities. Post-ATLASS follow up interventions helped laboratories to improve their detection capacity and increase the level of activity. Many of these laboratories would like to be re-assessed using the FAO-ATLASS. This study is a way to refine and adjust future interventions based on the AMR situation described and the feedback from countries and laboratories. FAO is committed to establish and maintain a global system supporting national efforts to regularly generate and disseminate reliable AMR data in food and agriculture enabling monitoring and surveillance of AMR at national, regional, and global levels to inform evidence-based decisions. Assessments are the first steps, but it is also very important to implement follow up interventions to strengthen the surveillance and laboratory activities. FAO will continue supporting countries in developing their AMR surveillance systems according to national priorities.

## Data Availability

The original contributions presented in the study are included in the article, further inquiries can be directed to the corresponding author.

## Footnotes

^1^ https://qgis.org/en/site/

^2^ https://www.fao.org/antimicrobial-resistance/resources/tools/fao-atlass/en/

^3^ https://new.amrcountryprogress.org/

^4^ https://new.amrcountryprogress.org/
