# Supplementary material for: Building capacities in Sub-Saharan African countries for antimicrobial resistance surveillance in the food and agriculture sectors using the FAO ATLASS tool
Source: Front Vet Sci. 2025 Nov 3;12:1607013. doi: 10.3389/fvets.2025.1607013 (PMC12621323; doi:10.3389/fvets.2025.1607013)
Supplement: Supplementary file 1 [file Data_Sheet_1.docx]

Supplementary Material

# Supplementary elements

## Structure of the ATLASS tool


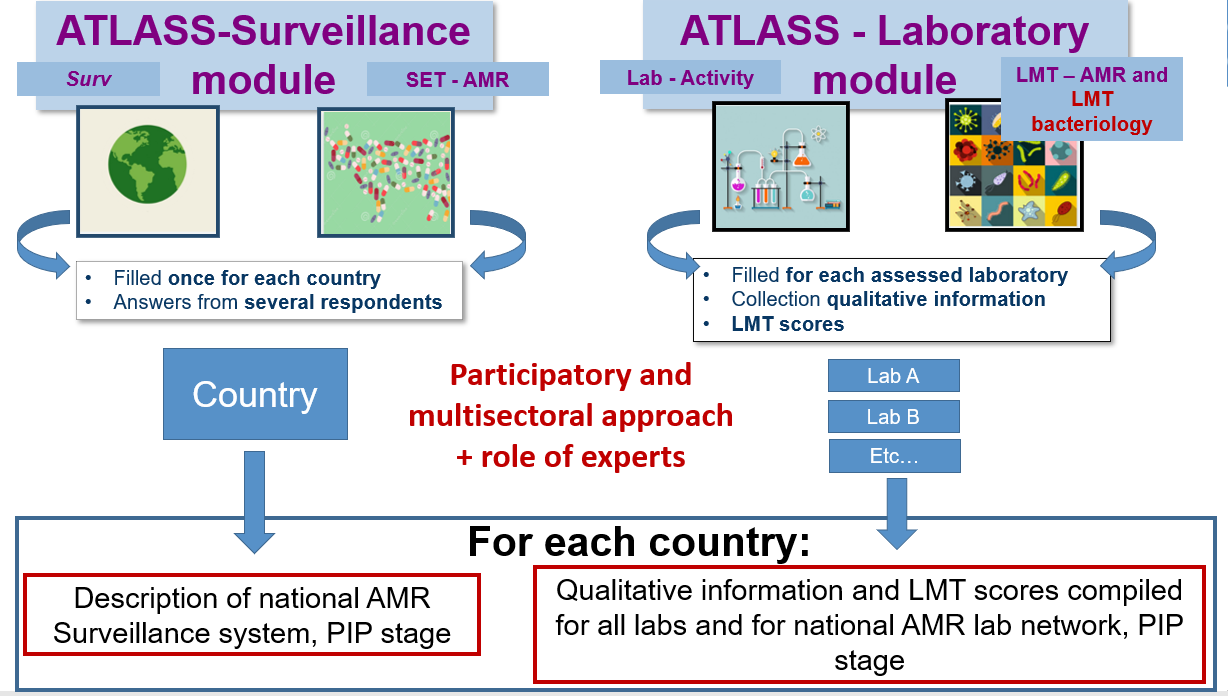


Figure 1: FAO-ATLASS structure


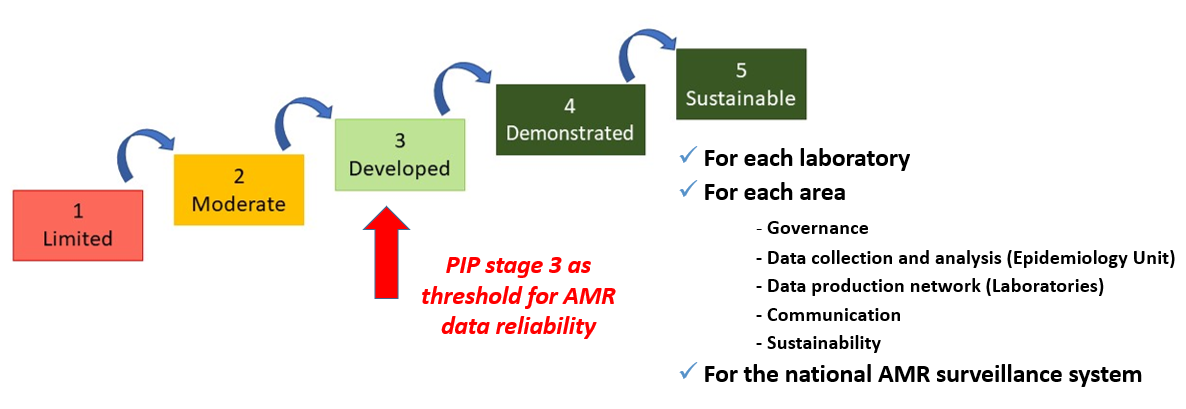


Figure 2: FAO-ATLASS Progressive Improvement pathway (PIP)

FAO-ATLASS has two main modules, ATLASS Surveillance and ATLASS Laboratory. Each module includes two questionnaires (Figure 1). In-country assessment missions, conducted by trained assessors, include meetings with key stakeholders and visits to selected laboratories involved in AMR surveillance to assess the performance of AMR-linked activities based on the five main areas. Based on the questionnaires’ answers, an ATLASS PIP stage is proposed (Figure 2). ATLASS PIP stages assist in making recommendations to governments to prioritize actions for improvement. A detailed description of the FAO-ATLASS tool and its features is available in the following article through the link <https://pubmed.ncbi.nlm.nih.gov/36825205/> (Keck et al., 2023).

## Post ATLASS assessment survey questionnaires

**Questionnaire for surveillance systems**

1. Country: ______________________________________
2. Sub-region: Eastern Africa – Central Africa – Southern Africa – Western Africa
3. Name of contact person:
4. Email of the contact person: __________________________________
5. How long has your surveillance system been in place? 1, 2, 3, more than 3 years
6. How many labs are involved in your AMR surveillance system?
7. Is there a national AMR surveillance strategy/programme/plan integrating components and implementation of activities across different AMR surveillance programs in food and agriculture? Yes or No
8. What is the status of development and implementation of this integrated strategy/programme/plan? (only one answer) __ ​Under development - ​Finalized but waiting for final endorsement - ​Approved by Government but no funds allocated - ​Approved by Government with allocated funds, and under implementation - ​I don’t know
9. What is (are) the scope (s) of your AMR surveillance system? (one or more) Animal health – Food Safety – Environment – Plant health
10. How would you define the method(s) used in your country for collection of samples for integrated AMR surveillance in food and agriculture? (one or more answers)

- Active (with an epidemiologically defined sampling framework)
- Passive (from clinical samples submissions, “collect what is available”)
- Comprehensive (all providers/laboratories)
- Sentinel/targeted (chosen to set of sites)
- Continuous (ongoing)
- Episodic (limited period)
- Enhanced (specific data collection in addition to routine/continuous surveillance) Specific projects

1. Do you have a formal Epidemiological unit dedicated to AMR? Yes or No
2. How many times has your system been assessed using FAO-ATLASS tool? 1, 2, 3, More than 3 times
3. Who did the assessments? self-assessment – External Assessment – Both
4. When was the last assessment? (Year, Month)
5. Does the PIP stage determined for your surveillance system reﬂect your stage in AMR surveillance? Yes – Partially – No
6. What is your general appreciation of the relevance of the FAO-ATLASS Assessment recommendations? Very useful – A bit useful – Not useful
7. Comments.? _____________________________________________________
8. Would you like to be reassessed using the FAO-ATLASS tool? Yes or No and why?
9. Did you receive FAO or another Partner support after the FAO-ATLASS Assessment of your surveillance system? Yes or No
10. If yes, specify the Partner (s).
11. What kind of support (one or more)?: Training – equipment – reagents – field missions for sample collection – EQA program - Other. ______________
12. In which domain did you observe a positive impact from the ATLASS assessment of the national surveillance system in your country?

- Implementation of a surveillance system? Yes or No, If yes, please comment
- Strengthening existing surveillance system? Yes or No, If yes, please comment
- Better Governance? Yes or No, If yes, please comment
- Better data collection and analysis? Yes or No, If yes, please comment
- Better data production? Yes or No, If yes, please comment
- Better communication? Yes or No, If yes, please comment
- Better sustainability? Yes or No, If yes, please comment
- Other impact? Please specify
- No positive impact? Yes or No; please comment

1. Do you have any success story to share? If yes please provide

**Questionnaire for laboratories**

1. Country : ______________________________________
2. Sub-region: Eastern Africa – Central Africa – Southern Africa – Western Africa
3. Name of the Laboratory : _____________________
4. Name of contact person:
5. Title/Job position and email of the contact person:
6. Are you a Central or Regional/Sub-national Laboratory in the country?
7. Are you involved in AMR detection? Yes/no.
8. Please specify the purpose (one or more): diagnostics, surveillance, research
9. If yes and you are supporting AMR surveillance, what is your main role? Sentinel/Field laboratory – national Reference laboratory?
10. If not, why?
11. What is your domain as a bacteriology lab? (one or more) Animal health – Food Safety – Environment – Plant Health-Public Health
12. How many times has your laboratory been assessed using FAO-ATLASS tool? 1, 2, 3, More than 3 times
13. Do you have FAO-ATLASS tool trained personnel in your Laboratory? Yes or No
14. If No, will you be interested ? Yes or No
15. Who did the assessments? self-assessment – External Assessment – Both
16. When was the last assessment? (Year, Month)
17. Does the PIP stage determined for your laboratory reﬂect your lab's stage in AMR detection? Yes – Partially – No
18. Please comment.
19. How do you rate the FAO-ATLASS Assessment recommendations? Very useful – A bit useful – Not useful
20. Please comment.
21. Would you like to be reassessed using the FAO-ATLASS tool? Yes or No
22. Why?
23. Is your laboratory participated in any AMR Proficiency Testing Scheme (EQA)? If yes, please indicate the organizing institution and the year
24. If not, why?
25. Did you receive FAO or another Partner support after the FAO-ATLASS Assessment of your laboratory for capacity building? Yes or No
26. If yes, specify the Partner (s).
27. What kind of support (one or more)?: Training – equipment – reagents – Lab buidling renovation-field missions for sample collection – EQA program - Other. ______________
28. In which domain did you observe a positive Outcome/impact from the ATLASS assessment in your laboratory?

- Increased commitment of the Lab top management in AMR activities? Yes or No
- Promoting national and international collaboration? Yes or No
- Establishing and/or strengthening the relationship within the national AMR laboratory network? Yes or No
- Staffing? Yes or No
- Improved skills of the personnel? Yes or No. If yes specify
- Increased capacity for diagnosis? Yes or No. If yes please specify
- Increased number of samples tested? Yes or No. If yes provide an estimated number of samples tested before and currently)
- Better customer-Laboratory relationship? Yes or No
- Quality assurance? Yes or No
- Other impact? Please specify
- If you lab has been reassessed using the ATLASS tool has the PIP increased?

1. Do you have a specific success story to share? If yes please provide

# Supplementary Figures and Tables

## Supplementary Figures


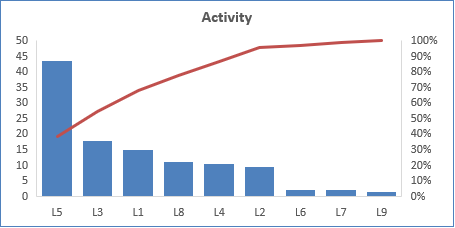

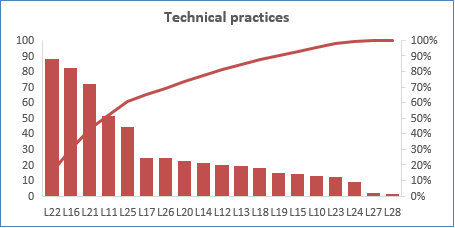


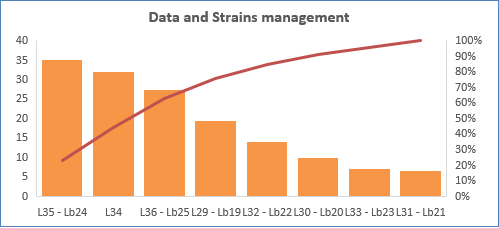

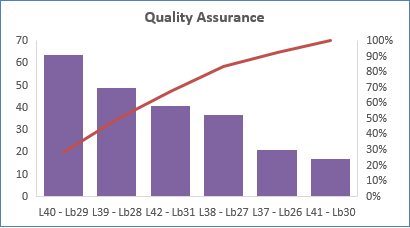


**Note**: See table 1 below for FAO-ATLASS factors corresponding to the code represented on the Pareto chart.

Figure 1: Pareto charts prioritizing the components to improve in different domains for all the laboratories assessed using FAO-ATLASS in Africa, 2018-2024


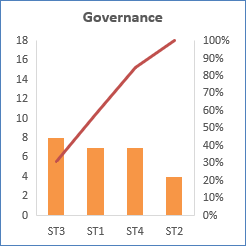

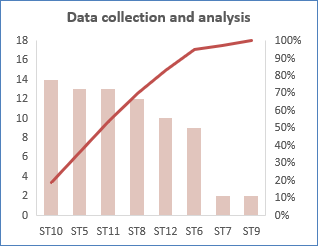

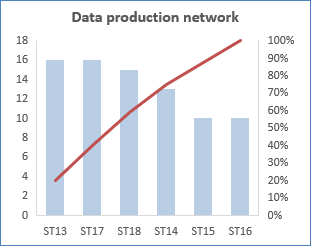

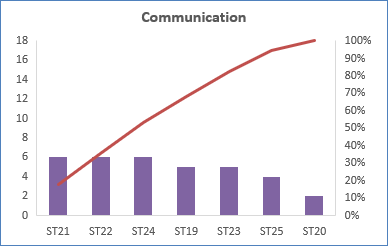

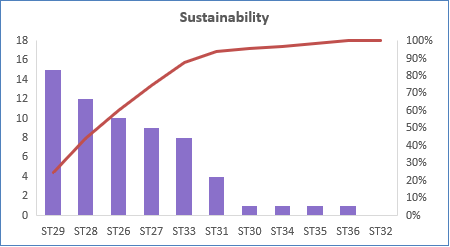


**Note**: See table 2 below for FAO-ATLASS factors corresponding to the code represented on the Pareto chart.

Figure 2: Pareto charts prioritizing the factors to improve in different domains for all surveillance systems assessed using FAO-ATLASS in Africa, 2018-2024

**Figure 4: Laboratory status and microbiology activities from the survey on ATLASS assessment outcomes.**

Figure 5: Distribution of laboratory assessments and date of the last assessment, by the time of the follow up survey

## Supplementary Tables

Table 1: FAO-ATLASS codes and subcategories within domains for laboratories

| **Code** | **Subcategories within domains for laboratories** |
| --- | --- |
| L1 | Financial capacity (allocation of funds) |
| L2 | Management |
| L3 | Quality of samples submitted |
| L4 | *Sharing of results with customers* |
| L5 | Sample acceptation criteria |
| L6 | Training about antimicrobial resistance |
| L7 | Scientific publications |
| L8 | Collaboration with other laboratories in the country |
| L9 | Collaboration with laboratories outside the country |
| L10 | Biosafety of Bacteriology laboratory |
| L11 | Equipment for bacteriology and AST |
| L12 | *ANIMAL HEALTH DISEASES - Media and consumable-* |
| L13 | *FOOD SAFETY - Media and consumable* |
| L14 | *WATER and ENVIRONMENT - Media and consumable* |
| L15 | *PLANT HEALTH - Media and consumable* |
| L16 | Reagents availability for AST or identification |
| L17 | Bacteriology methods |
| L18 | Bacterial identification |
| L19 | Standard for AST |
| L20 | Bacterial inoculum calibration for AST |
| L21 | Panels definition |
| L22 | Revision of panels of antibiotics |
| L23 | *Method for reading disk diffusion results* |
| L24 | *Method for reading MIC results* |
| L25 | *Standard for interpretation of disk diffusion results* |
| L26 | *Standard for interpretation of MIC results* |
| L27 | Molecular characterization (resistance gene confirmation or typing) |
| L28 | Sequencing of resistant strains |
| L29 - Lb19 | Sample identification and follow-up |
| L30 - Lb20 | Proportion of isolates archived in a library |
| L31 - Lb21 | Method for bacterial preservation |
| L32 - Lb22 | Inventory of archived isolates |
| L33 - Lb23 | Duration of bacterial isolates archiving |
| L34 | *Individual reports on AMR data to the customers* |
| L35 - Lb24 | Data archiving |
| L36 - Lb25 | *AMR data transmission to a dedicated epidemiology unit (if existing) OR strains transmission* |
| L37 - Lb26 | SOPs on AMR detection implemented OR BACT |
| L38 - Lb27 | SOPs on AMR detection updating OR BACT |
| L39 - Lb28 | Reference strains for AST quality control OR BACT |
| L40 - Lb29 | Proficiency testing for AST OR BACT |
| L41 - Lb30 | Initial training in AMR testing OR BACT |
| L42 - Lb31 | Staff skills validation and continuous proficiency OR BACT |

Table 2: FAO-ATLASS codes and components within domains for surveillance systems

| **Codes** | **Subcategories within domains for surveillance systems** |
| --- | --- |
| ST1 | Existence of an operational structure representative of the stakeholders involved in AMR surveillance under One Health approach (multi-sectoral working group(s) or coordination committee on AMR) |
| ST2 | Development of a National Action Plan on AMR involving the food and agriculture sectors |
| ST3 | Relevance of AMR surveillance objectives and AMR indicators in food and agriculture sectors |
| ST4 | Regulations on AMR surveillance organization in the food and agriculture sectors |
| ST5 | Existence of an operational management structure (central epidemiology unit) in food and agriculture sectors |
| *ST6* | *Frequency of coordination meetings between central epidemiology unit with local units* |
| ST7 | Representativeness of the surveillance sampling scheme in food and agriculture sectors including environment |
| ST8 | Adequate skill level in AMR epidemiology of members of the central unit |
| ST9 | Adequacy of the data management system for the needs of the AMR surveillance system (database, etc.) |
| ST10 | Data input interval in accordance with the objectives and use of AMR surveillance system results |
| ST11 | AMR data verification and validation procedures formalized and operational |
| ST12 | Analysis of AMR data fits the needs of the system |
| ST13 | Effective integration of competent laboratories in the AMR surveillance system |
| ST14 | Level of the standardization of work between different laboratories involved in the AMR surveillance system |
| ST15 | Relevance of laboratory diagnostic techniques |
| ST16 | Technical level of AMR data management of the laboratory network |
| ST17 | Frequency of data transmission to the epidemiology unit |
| ST18 | Harmonization of data transmitted to the epidemiology unit |
| ST19 | External policy for communication with decision makers and other stakeholders |
| ST20 | Identification and coverage of key stakeholders’ expectations about the results of the surveillance system |
| ST21 | Existence of awareness building AMR programs for surveillance actors |
| ST22 | Communication of risk assessment outcomes to relevant parties |
| ST23 | Regular release of reports on AMR surveillance results |
| ST24 | Systematic distribution of AMR surveillance results to field actors (outside of a report) |
| ST25 | Presence of a communication system organized between field actors (mail, websites, telephone…) |
| ST26 | Adequacy of material and financial resources for the multi-sectoral working group(s) or coordination committee on AMR |
| ST27 | Adequacy of financial resources for the implementation of the National AMR action plan |
| ST28 | Adequacy of human, material, and financial resources for AMR data production (laboratory network) needs |
| ST29 | Adequacy of human, material, and financial resources for AMR data collection and analysis (epidemiology) needs |
| ST30 | Adequacy of human, material, and financial resources for communication needs |
| ST31 | Regular advanced training for actors of the surveillance |
| ST32 | Adequacy of material and financial resources for training |
| ST33 | Development and validation of performance indicators for the AMR surveillance system |
| ST34 | Regular measurement, interpretation, and dissemination of performance indicators |
| ST35 | External assessment carried out |
| ST36 | Implementation of corrective measures |
